# Clinical impact of very high‐power‐short‐duration catheters on biomarkers after atrial fibrillation ablation

**DOI:** 10.1002/joa3.70060

**Published:** 2025-04-09

**Authors:** Andrea Bernardini, Alessandro Paoletti Perini, Cristiano Salvatore Zaccaria, Davide Ciliberti, Umberto Signorini, Francesco Grossi, Raffaele Martone, Serena Fatucchi, Alenja Bertini, Anna Arretini, Lisa Innocenti, Irene Capecchi, Margherita Padeletti, Massimo Milli, Andrea Giomi

**Affiliations:** ^1^ Cardiology and Electrophysiology Unit, Department of Medical Specialties Azienda USL Toscana Centro, Santa Maria Nuova Hospital Florence Italy; ^2^ Department of Experimental and Clinical Medicine University of Florence Florence Italy

**Keywords:** atrial fibrillation, BNP, pulmonary vein isolation, radiofrequency, vHPSD

## Abstract

**Background:**

Very high‐power short‐duration (vHPSD) catheters are associated with less irrigation fluid load than standard (STD RF) ablation catheters. However, the impact of this fluid reduction on biomarkers in pulmonary vein isolation (PVI) for atrial fibrillation (AF) remains unknown.

**Methods and Aim:**

Biomarkers of heart failure, myocardial injury, and systemic inflammation status as Brain Natriuretic Peptide (BNP), high‐sensitivity Troponin I (hsTnI), and C‐reactive protein (CRP) were collected pre‐ and post‐procedure of PVI for symptomatic AF. The study aimed to assess the impact of vHPSD catheter compared to an STD catheter (respectively irrigation of 8 vs. 15 mL/min during ablation) on biomarker alterations.

**Results:**

The study included 83 consecutive patients (59 males [71.1%], mean age 62.6 ± 11 years), with vHPSD catheters used in 53 cases (63.9%). No significant baseline differences were observed between groups.Fluid irrigation resulted in significantly lower with vHPSD catheter than STD RF (434.8 ± 105.6 vs. 806.6 ± 256.5 mL, *p* < .001). Correspondingly, BNP variation was significantly lower in the vHPSD group than in the STD RF group, both in absolute change (12 [IQR −9–47] pg/mL vs. 44.5 [IQR 21–88.7] pg/mL, *p* = .002) and percentage change (16.3 [IQR −13.2–108.6] % vs. 84.1 [IQR 32.5–172.1] %, *p* = .012). When considering absolute values, a statistically significant increase in BNP was found only in the STD catheter group (from 52 [IQR 35.2–113.5] to 113 [IQR 66.7–189.5] pg/mL, *p* < .001), whereas no significant increase was observed in the vHPSD group (*p* = .06). CRP levels increased post‐PVI in both groups, but the delta was significantly lower in the vHPSD group (*p* = .025). No significant differences in post‐procedural hsTnI were detected between groups.

**Conclusion:**

The use of a vHPSD catheter is associated with reduced fluid irrigation and a correspondingly smaller increase in BNP, a biomarker indicative of fluid overload and heart failure.

## Graphical abstract

Differences in fluid irrigation (A), pre‐ and post‐procedure BNP absolute values change (B), and BNP percentage values change (C) in standard catheters (STD RF) versus very high‐power short‐duration catheters (vHPSD). vHPSD catheters use is associated with significantly less irrigation fluid than with standard STD RF catheters during pulmonary vein isolation for atrial fibrillation. This reduced fluid load is associated with a significantly smaller increase in BNP levels, suggesting a lower impact on fluid overload and reducing the risk of heart failure.

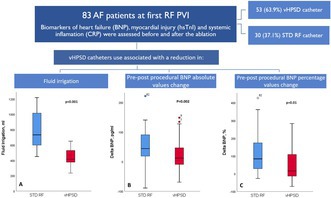

## 1 INTRODUCTION

Radiofrequency catheter ablation represents a cornerstone in the treatment of symptomatic atrial fibrillation (AF),^1^ and PVI (pulmonary vein isolation) is recommended as a first‐line option to reduce symptoms, recurrence, and progression of AF.^2^ In particular, an improvement in clinical outcomes and mortality is also observed in patients with heart failure, in which the fluid balance represents a major determinant of symptoms and prognosis.^2^, ^3^, ^4^ On the contrary, the technology of RF catheters evolved in recent years with the introduction of very high‐power short‐duration (vHPSD) catheters: these catheters enable an optimized lesion geometry, maintaining comparable safety and long‐term rhythm outcomes while also shortening procedure duration.^5^, ^6^, ^7^ Besides, vHPSD catheters are associated with less irrigation fluid load than standard radiofrequency (STD RF) ablation catheters.^8^, ^9^, ^10^, ^11^ However, the effect of this fluid reduction and the different energy dosages on biomarker levels following PVI for AF remains unclear. This study aims to evaluate the impact of using a vHPSD catheter compared to an STD RF one, on variations in Brain Natriuretic Peptide (BNP), high‐sensitivity Troponin I (hsTnI), and C‐reactive protein (CRP) as biomarkers of heart failure, myocardial injury, and systemic inflammation, respectively.

## 2 METHODS

### 2.1 Study population

Our retrospective observational study included 83 consecutive patients referred to our electrophysiological lab for PVI because of symptomatic paroxysmal (PAF) or persistent atrial fibrillation (PsAF), from Sept 2023 to October 2024. All patients in the study had an indication of transcatheter ablation of AF according to current guidelines.^2^ The patients were stratified into two groups according to the catheter ablation used (standard RFA vs. vHPSD). The ablation modality was selected based on available technology and/or the operator's preference. For the purpose of this study, we considered only patients at their first ablation procedure and without known structural heart diseases. Only patients that had PVI with a wide area circumferential ablation (WACA) lesion were considered, excluding patients that underwent additional lines in the right or left atria or Marshall vein ethanol infusion, to avoid potential biases in biomarkers modifications between the two populations. The definitions of PAF or PsAF and the clinical indications to proceed to ablation were given according to the latest ESC Guidelines.^2^ All patients underwent a careful cardiovascular risk factors assessment and a transthoracic echocardiography. The study complies with the Declaration of Helsinki, and all participants provided written informed consent.

### 2.2 Blood sampling

Brain Natriuretic Peptide (BNP), high‐sensitivity Troponin I (hsTnI), C‐reactive protein (CRP), creatinine, hematocrit, electrolyte levels, and white blood cell count, including neutrophil count, were collected both before and after the procedure. Serum high‐sensitivity cardiac Troponin I (hs‐cTnI) was measured using the Elecsys Troponin I high‐sensitive assay (cut‐off value of 11.6 pg/mL, Roche Diagnostics). The assay‐specific cut‐off value for CRP was set at 0.5 mg/dL. For BNP (Roche Diagnostics Immunoassay), values inferior to 35 pg/mL were considered unlikely for heart failure, according to the latest ESC Heart Failure Consent document.^12^ BNP evaluation was preferred over NT‐proBNP because of its shorter half‐life,^13^ allowing for more precise stratification of biomarker changes over short time intervals. Additionally, BNP is less influenced by renal disease stage compared to NT‐proBNP,^14^ while maintaining comparable diagnostic and prognostic accuracy.^12^ Blood collection from an antecubital vein was performed in the afternoon of the day before the procedure and in the early morning of the day after the ablation procedure. As PVI procedures at our center are consistently scheduled as the first cases of the day, no significant variation was observed in the interval between the ablation and subsequent blood collection. Biomarkers were therefore measured approximately 13 hours before and 19 h after the PVI.

### 2.3 Electrophysiological study and ablation

During the EP study, patients were monitored by 12‐lead ECG, noninvasive blood pressure measurements, and pulse oximetry. At the beginning of the procedure, transesophageal or intracardiac echocardiography was performed to exclude left auricular thrombosis and to guide the transeptal puncture. The procedures were executed in mild sedation. The infusion of saline solution was limited to the minimum necessary during the procedure (less than 10 drops/min) and was carefully avoided in the post‐procedural phase. Diuretic medications were discontinued postoperatively, except in cases of urgency. Additionally, none of the patients were receiving sacubitril/valsartan therapy. Intracardiac access was obtained via venous femoral accesses: a deflectable decapolar catheter (Decanav, Biosense Webster, Diamond Bar, CA) was inserted into the coronary sinus, and a multipolar mapping catheter (Pentaray or Octaray, Biosense Webster, Diamond Bar, CA) was used to map the left atrium. Ablation was performed with radiofrequency energy: standard power‐standard duration (3.5 mm open irrigated‐tip catheter, Thermocool SF, Biosense Webster, Diamond Bar, CA, saline infusion rate during ablation 15 mL/min) or vHPSD (high‐energy, temperature‐controlled ablation catheter QDOT Micro, Biosense Webster, Diamond Bar, CA, saline infusion rate during ablation 8 mL/min in QMODE+, irrigation rate dependent on temperature during QMODE). To aid ablation catheter manipulation and stabilization, a steerable long sheath (Agilis, Abbott, Chicago, IL or CARTO VIZIGO, Biosense Webster, Diamond Bar, CA) was used. After reaching the left atrium by the transeptal puncture, heparin (100 IU/kg) was administered to reach an activated clotting time of more than 300 s. In all patients, a WACA lesion was performed. For the STD RF group, the lesions were created based on the ablation index criteria^15^, ^16^ (CLOSE protocol): a power‐controlled mode with 30 W was used at the posterior PV wall and 35 W at the anterior PV wall, reaching an Ablation Index target of 400 for the posterior side and 550 for the anterior one with an interlesion distance <6 mm. In the vHPSD group, an energy of 90 W for 4 s with an interlesion distance <6 mm was delivered for the lesion creation in the PV posterior wall; for the anterior PV wall, we used the same approach as for the STD RF group (power‐controlled mode, 35 W, reaching an ablation index of 550, interlesion distance <6 mm). A bidirectional block from the pulmonary vein was assessed after the ablation. A careful remap with the multipolar mapping catheter was executed after the ablation to assess eventual gaps in thelesion.

### 2.4 Statistical analysis

Continuous variables were expressed as mean and standard deviation (SD) in case of normally distributed data, or median and 25%–75% interquartile range (IQR) for non‐normally distributed data. Categorical variables were annotated as number (*n*) and percentage (%). Normally distributed data was assessed by the Shapiro‐Wilk test. For continuous variables and independent samples, comparison between groups was evaluated using the unpaired *t*‐test for normally distributed variables or Mann–Whitney *U* test for non‐normally distributed data. Pearson *χ* ^2^ test was applied for categorical variables. In the case of paired samples, for continuous variables, comparison between groups was evaluated using paired *t*‐test for normally distributed variables or Wilcoxon signed rank test for non‐normally distributed data, while McNemar's test was applied for categorical variables. A *p*‐value <.05 was considered statistically significant. Statistical analysis was performed with SPSS Statistics version 25 (IBM Corporation).

## 3 RESULTS

**FIGURE 1:**
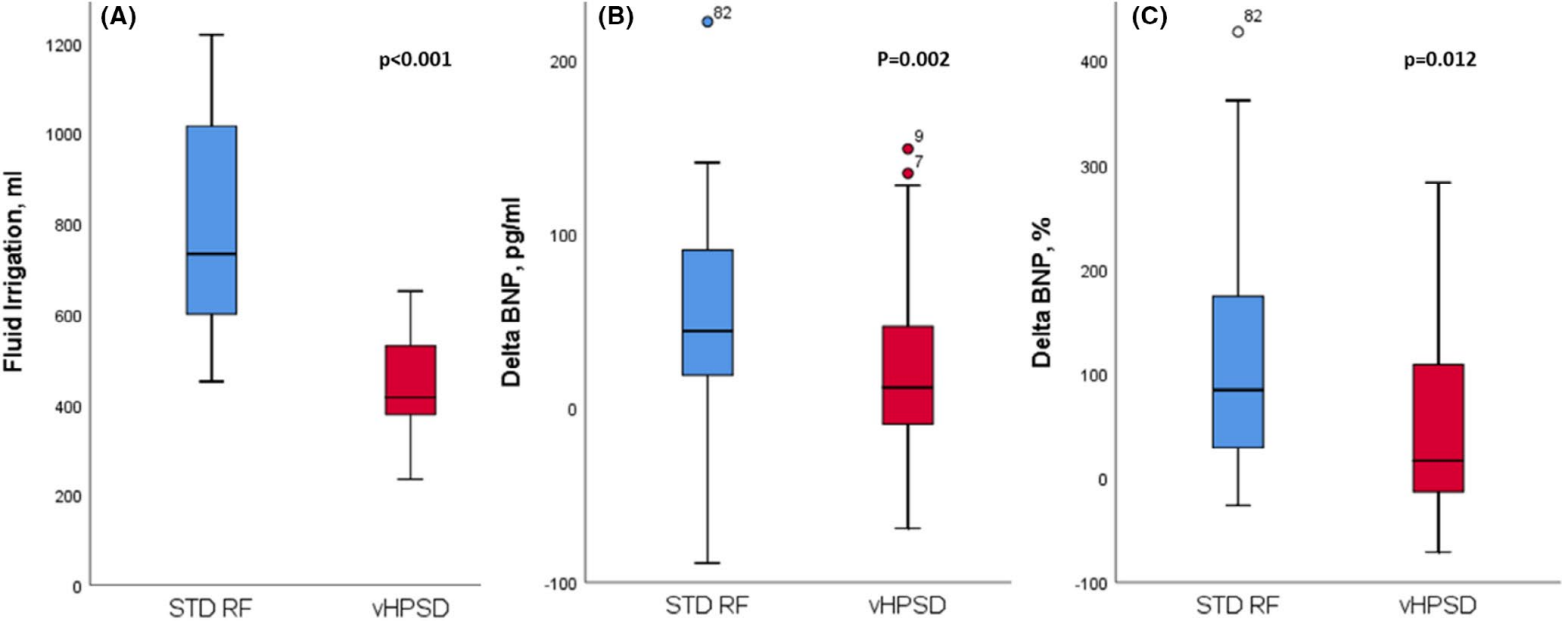
Box plots comparing fluid irrigation (A), pre‐ and post‐procedure absolute value changes (B) and percentage value changes (C) in standard catheters (STD RF) and very high‐power short‐duration catheters (vHPSD).

**FIGURE 2:**
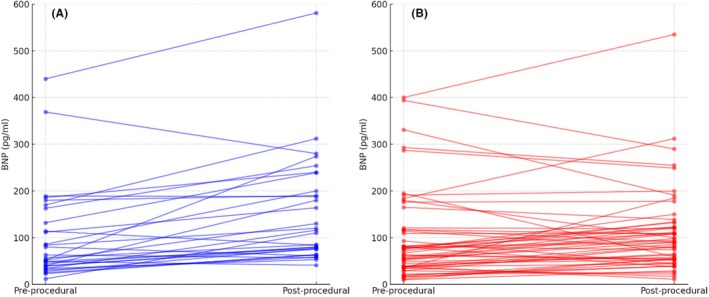
Pre–post‐procedural BNP absolute values change in every single patient using standard RF ablation catheters (A) and vHPSD catheters (B).

The study included 83 consecutive patients (59 males [71.1%]) with a mean age of 62.6 ± 11 years. The vHPSD catheter for ablation was used in 53 (63.9%). PAF was the most frequent form of AF (70 patients [84.6%]) No statistically significant baseline differences were detected between the two groups (Table 1). Fluid irrigation resulted significantly lower using the vHPSD catheter than STD RF one (434.8 ± 105.6 vs. 806.6 ± 256.5 mL, *p* < .001) with a comparable number of lesions between the two groups. Correspondingly, BNP variation (Figures 1 and 2, Table 3) was significantly lower in the vHPSD group than in the STD RF group, both in absolute change (12 [IQR −9–47] pg/mL vs. 44.5 [IQR 21–88.7] pg/mL, *p* = .002) and percentage change (16.3 [IQR −13.2–108.6] % vs. 84.1 [IQR 32.5–172.1] %, *p* = .012). When considering absolute values (Table 2), a statistically significant increase in BNP was found only in the STD catheter group (from 52 [IQR 35.2–113.5] to 113 [IQR 66.7–189.5] pg/mL, *p* < .001), whereas no significant increase was observed in the vHPSD group (from 67 [IQR 38–117] to 90 [IQR 54–137] pg/mL, *p* = .06). Given the values of BNP below 35 pg/mL considered unlikely for heart failure,^10^ 63 patients (75.9%) had a pre‐procedural BNP value above this threshold. Also in these patients, the variation in BNP levels before and after the procedure was greater in the STD catheter group compared to the vHPSD group, both in absolute and percentage changes (*p* = .009, *p* = .028 respectively, Table 3). Furthermore, when examining pre‐and post‐procedural absolute values, a statistically significant increase in BNP was observed only in the STD catheter group (*p* = .003), while no significant increase was noted in the vHPSD group (*p* = .35, Table 2).

**TABLE 1:** Baseline and procedural characteristics of the study population.

Variables	All patient (*N* = 83)	STD RF catheter (*N* = 30)	vHPSD catheter (*N* = 53)	*p*‐value
Age, year	62.6 ± 11	65.7 ± 10.9	60.9 ± 10.8	.60
Male, *n* (%)	59 (71.1%)	21 (70%)	38 (71.7%)	.53
BMI, kg/m^2^	26.7 ± 4.4	27.4 ± 5.4	26.1 ± 3.3	.2
Hypertension, *n* (%)	41 (49.4%)	16 (53.3%)	25 (47.2%)	.38
Diabetes, *n* (%)	11 (13.3%)	4 (13,3%)	7 (13,2%)	.62
Obesity, *n* (%)	15 (18.1%)	9 (30%)	6 (11.3%)	.12
Dyslipidemia, *n* (%)	34 (41%)	13 (43.3%)	21 (39.6%)	.48
Smokers, *n* (%)	17 (20.5%)	7 (23.3%)	10 (18.9%)	.41
Former smokers, *n* (%)	26 (31.3%)	11 (36.7%)	15 (28.3%)	.29
OSAS, *n* (%)	9 (10.8%)	3 (10%)	6 (11.3%)	.58
CAD, *n* (%)	7 (8.4%)	2 (6.7%)	5 (9.4%)	.44
PAF, *n* (%)	74 (89.2%)	26 (86.7%)	48 (90.6%)	.64
PsAF, *n* (%)	9 (10.8%)	4 (13.3%)	5 (9.4%)	.62
LVEF, %	57.5 ± 5.6	58.4 ± 6.1	57 ± 5.4	.44
LA area, cm^2^	24.1 ± 8.4	23.5 ± 3.8	24,4 ± 7.3	.75
Mitral Regurgitation^a^, *n* (%)	4 (4.8%)	2 (6.7%)	2 (3.8%)	.60
AF at beginning of procedure, *n* (%)	13 (15.7%)	5 (16.7%)	8 (15.1%)	.45
Pre‐procedural high‐BNP^b^, *n* (%)	63 (76%)	22 (73.3%)	41 (77.4%)	.43
Procedure duration, min	157.3 ± 34.1	176.8 ± 36.2	144.8 ± 26.5	.**02**
Fluoro time, s	562.1 ± 343.3	554.2 ± 479.4	566.8 ± 240.2	.9
Fluoro dose, Gy*cm^2^	16.7 ± 17.2	18 ± 22.9	15.9 ± 13.3	.7
RF time, s	1511.2 ± 678.3	1873.5 ± 768.7	1288.2 ± 514.1	.**005**
Fluid irrigation, ml	576.4 ± 253.4	. 806.6 ± 256.5	434.8 ± 105.6	.**001**
Fluid irrigation + procedural saline infusion	657 ± 257.8	894.6 ± 256.5	510.8 ± 105.6	.**001**
Number of lesions, *n*	79.6 ± 18.4	78.5 ± 11.5	80.1 ± 16.	.76
Energy Dose, kJ	64.1 ± 52.9	73.9 ± 24.6	57.8 ± 64.6	.35

*Note*: Data are presented as: Mean ± Standard deviation or n (percentage). *p* < .05 considered as statistically significant. Statistically significant differences are enhanced in bold.

Abbreviations: BMI, body mass index; BNP, brain natriuretic peptide; CAD, coronary artery disease; LA, left atrium; LVEF, left ventricular ejection fraction; OSAS, obstructive sleep apnea syndrome; PAF, paroxysmal atrial fibrillation; PsAF, persistent atrial fibrillation; RF, radiofrequency; STD RF, standard radiofrequency catheter; vHPSD, very high power short duration.

^a^ Intended as more than mild.

^b^ Considering as cutoff for High‐BNP as 35 pg/mL.

**TABLE 2:** Laboratory results of the study population.

Variables	Pre‐PVI laboratory test		* *p*‐value	Post‐PVI laboratory test		* *p*‐value	** *p*‐value	
	STD RF catheter (*N* = 30)	vHPSD catheter (*N* = 53)		STD RF catheter (*N* = 30)	vHPSD catheter (*N* = 53)		STD RF catheter (*N* = 30)	vHPSD catheter (*N* = 53)
WBC, 10^9^/L	7.8 (6.5–9.3)	6.9 (5.5–8.8)	.12	10.1 (8.3–11.4)	9.1 (7.3–11.4)	.88	.**005**	**<.001**
Neutrophils, 10^9^/L	5.4 (3.4–6.6)	4.2 (3.1–5.6)	.07	6.9 (5.2–8.1)	5.5 (4.5–7.4)	.17	.**03**	**<.001**
Hematocrit, %	41.6 (38.4–44)	43 (40–45)	.21	40 (37.1–42.3)	41 (38–45)	.36	.**02**	.**02**
Creatinine, mg/dL	0.92 (0.8–1.2)	0.9 (0.8–1.2)	.4	0.82 (0.7–1.03)	0.89 (0.8–1.1)	.25	**<.001**	**<.001**
Sodium, mEq/L	140 (138.2–141.7)	142 (141–145)	.08	139 (138.2–142)	140 (139–142)	.25	.26	**<.001**
Potassium mEq/L	4.4 (4.2–4.7)	4.3 (4–4.6)	.24	3.8 (3.5–4.1)	4 (3.8–4.2)	.06	**<.001**	**<.001**
CRP, mg/mL	0.13 (0.07–0.21)	0.17 (0.07–0.21)	.91	1.2 (0.7–2.3)	1.1 (0.6–1.3)	.32	**<.001**	**<.001**
BNP, pg/mL	52 (35.2–113.5)	67 (38–117)	.67	113 (66.7–189.5)	90 (54–137)	.67	**<.001**	.06
High‐BNP population^a^, pg/mL	82 (49–139.7)	80 (55–170.5)	.67	147 (82.2–239.2)	108 (64–136.5)	.16	.**003**	.35
Hs‐TnI, pg/mL	4.5 (3.5–6.7)	5 (3.1–7.6)	.39	1757.5 (1275.4–2214.9)	2122.8 (1542–2577.1)	.11	**<.001**	**<.001**

*Note*: Statistically significant differences are enhanced in bold.

Abbreviations: BMI, body mass index; BNP, brain natriuretic peptide; CRP:C‐reactive protein; hsTnI, High‐sensitivity cardiac Troponin I; PVI, pulmonary vein isolation; STD, standard; vHPSD, very high‐power short duration; WBC, white blood cell.

^a^ Considering as cutoff for elevated BNP as 35 pg/mL.

* *p*‐value of comparison between pre‐ and post‐procedural values of vHPSD versus STD RF catheters.

** *p*‐value of comparison between pre and post‐procedural values with the same catheter.

**TABLE 3:** Absolute and percentage changes in laboratory tests of the study population.

Variables	STD RF catheter (*N* = 30)	vHPSD catheter (*N* = 53)	*p*‐value
WBC, 10^9^/L	1.74 (−0.07–4.7)	2.5 (1.1–5.05)	.59
WBC, %	29.2 (−0.7–69.4)	38 (15.5–63.6)	.38
Neutrophils, 10^9^/L	1.6 (−0.6–3.9)	1.8 (−0.16–3.7)	.96
Neutrophils, %	30.9 (−10.4–101.4)	54.2 (−3.6–108.3)	.58
Hematocrit, %	−2 (−4.8 – −0.02)	−1.7 (−4–1.1)	.36
Hematocrit, %	−4.6 (−10.9–0.07)	−4.1 (−9.1–2.3)	.33
Creatinine, mg/dL	−0.04 (−0.13 – −0.02)	‐0.02 (−0.11 – −0.02)	.14
Creatinine, %	−5.2 (−14,1 – −2.1)	−2.3 (−12.1 – −1.5)	.08
Sodium, mEq/L	‐1.5 (−3–2.7)	−2 (−4–0.1)	.17
Sodium, %	−1.06 (−2.1–1.97)	−1.4 (−2.7–0.1)	.13
Potassium, mEq/L	−0.45 (−0.71 – −0.3)	−0.26 (−0.7–0.1)	.**01**
Potassium, %	−10.4 (−16.3 – −6.6)	−5.7 (−16.3–0.1)	.**009**
CRP, mg/mL	1.01 (0.5–2.1)	0.85 (0.5–1.25)	.**025**
CRP, %	894.3 (300–1563.7)	600 (304.8–1500)	.68
BNP, pg/mL	44.5 (21–88.7)	12 (−9–47)	.**002**
BNP, %	84.1 (32.5–172.1)	16.3 (−13.2–108.6)	.**012**
High‐BNP group^a^, pg/mL	44 (−2–45)	7 (−26–46)	.**009**
High‐BNP group^a^, %	52.4 (−2.7–154)	5 (−15–70)	.**028**
Hs‐TnI, pg/mL	1754.2 (1271.3–2210.8)	2118.6 (1532–2573.3)	.08
Hs‐TnI, %	42387.6 (24232.8–60077.4)	45080.8 (25005.3–67718.4)	.74

*Note*: Data are presented as: Median (Interquartile range: 25th and 75th percentiles). *p* < .05 considered statistically significant. Statistically significant differences are enhanced in bold.

Abbreviations: BMI, body mass index; BNP, brain natriuretic peptide; CRP:C‐reactive protein; Hs‐TnI, high‐sensitivity cardiac Troponin I; PVI, pulmonary vein isolation; STD, standard; vHPSD, very high‐power short duration; WBC, white blood cell.

^a^ Considering as cutoff for elevated BNP as 35 pg/mL.

An increase in CRP values after PVI was observed in both groups (from 0.13 [IQR 0.07–0.21] to 1.2 [IQR 0.7–2.3] mg/dL, *p* < .001 for STD; from 0.17 [IQR 0.07–0.21] to 1.1 [IQR 0.6–1.3] mg/dL, *p* < .001 for vHPSD). However, a statistically significant difference was found in the change in CRP levels between the STD and vHPSD catheter groups (1.01 [IQR 0.5–2.1] mg/dL for STD vs. 0.85 [IQR 0.5–1.25] mg/dL for vHPSD, *p* = .025). A significant hsTnI increase was found in both groups after the PVI (Table 2, *p* < .001). However, although a trend in favor of an increased release of hsTnI after PVI associated with vHPSD catheters use, a statistically significant difference was not reached in terms of absolute or percentage change, or in absolute values after the procedure (respectively: from 1754.2 [IQR 1271.3–2210.8] to 2118.6 [IQR1532–2573.3] pg/mL, *p* = .08; 42387.6 [IQR 24232.8–60077.4] vs. 45080.8 [IQR 25005.3–67718.4] %, *p* = .74; 1757.5 [IQR 1275.4–2214.] vs. 2122.8 [IQR 1542–2577.1] pg/ml, *p* = .11). In both populations, a tendency to hemodilution was observed with a reduction in values of hematocrit, creatinine, and electrolytes. However, no significant differences in pre‐ and post‐procedure changes were found between the two groups, except for a more pronounced decrease of potassium level both in absolute and percentage changes in STD RF than in vHPSD (−0.45 [IQR −0.71 to −0.3] mEq/L vs. −0.26 [IQR −0.7 to 0.1] mEq/L, *p* = .01, and −10.4 [IQR −16.3 to −6.6] % vs. −5.7 [IQR −16.3 to −0.1]%, *p* = .009, respectively).

## 4 DISCUSSION

Our study systematically evaluated the effects of vHPSD catheter use on biomarkers associated with heart failure, systemic inflammation, and myocardial injury in a cohort of AF patients undergoing PVI. The main findings of our study are: (I) the use of a vHPSD catheter is associated with reduced fluid irrigation and a correspondingly smaller increase in post‐procedural BNP, a biomarker indicative of fluid overload and heart failure; (II) this difference is independent of pre‐procedure BNP levels; (III) vHPSD catheters are associated with a smaller CRP increase compared to STD RF catheters.

### 4.1 Fluid infusion and BNP changes

BNP is a hormone produced by cardiac myocytes in response to myocardial wall stress.^17^, ^18^ Elevated BNP levels indicate underlying cardiac dysfunction and have been linked to both the incidence of AF and its recurrence after catheter ablation.^19^, ^20^ AF induces significant hemodynamic derangements and structural remodeling, leading to elevated BNP levels. Specifically, AF causes hemodynamic load and wall stress because of loss of atrial kick, variability of stroke volume because of irregular ventricular rhythm, and loss of atrioventricular synchrony, all contributing to BNP elevation.^17^, ^21^ Furthermore, AF‐induced structural remodeling perpetuates a vicious cycle further elevating plasma BNP levels, particularly in patients with lower LVEF.^22^ In other words, elevated BNP levels are linked to an increased risk of AF because of shared factors such as myocardial fibrosis, ventricular dysfunction, and increased filling pressures.^17^, ^18^ But BNP also serves as a marker of fluid overload,^23^, ^24^ with its concentrations rising in response to volume and pressure overload in the heart, producing diuretic, natriuretic, and antihypertensive effects.^24^, ^25^ Fluid overload is a major determinant of symptoms in heart failure patients, and it is strongly associated with short‐ and long‐term prognosis.^4^, ^26^ It reflects the severity of the disease by indicating the depletion of neurohormonal compensatory mechanisms, while the congestion itself mechanically affects critical organs such as the heart, liver, kidneys, and lungs.^4^, ^27^ Congestive heart failure because of fluid overload after catheter ablation for AF is a reported complication.^28^, ^29^, ^30^, ^31^ The reduced fluid irrigation associated with vHPSD catheters mitigates this overload, as suggested in our study, by directly reducing BNP increase, a heart failure marker. This result appears independent of the pre‐procedural BNP values. Besides, the adoption of more extensive ablation strategies beyond PVI with consequent longer procedural times and number of lesions could amplify this effect. To our knowledge, this is the first study to evaluate changes in a heart failure biomarker in alignment with the fluid infusion characteristics associated with catheter use.

### 4.2 Inflammation and myocardial injury biomarkers changes

Patients undergoing radiofrequency ablation for AF manifest an inflammatory response within the first 3 days. Myocardial injury following RFA generates a reversible inflammatory reaction, which was directly correlated in a previous study by Lim et al.^32^ to early AF recurrences. However, differences in the extent of inflammation induced by vHPSD versus STD catheters have not been described to date. A recent paper by Popa et al.^7^ demonstrated a 60% reduction in WBC count following pulsed‐field ablation (PFA) compared to RF application, but without statistically significant differences between vHPSD or STD RF catheters. However, no measurements of CRP (a more accurate systemic inflammation biomarker than WBC^33^) were reported pre‐ and post‐procedure. In our study, a difference in CRP increase was associated with STD RF catheter use: vHPSD catheters create larger but shallower lesions, reducing the conductive heating phase,^34^ probably reducing thermal injury and the correlated inflammation in adjacent structures, such as the esophagus or pericardium.^7^

Troponin release, a marker of myocardial injury, has been investigated in previous studies involving RF catheters, with conflicting results regarding its association with arrhythmia recurrence.^7^, ^35^, ^36^ Although increased troponin release is known to be higher with vHPSD catheters compared to STD RF ones,^7^ our study observed only a trend toward a greater troponin increase with vHPSD catheters. The increase did not reach statistical significance, possibly because of the vHPSD approach being confined exclusively to the posterior LA wall. This limitation reduced the overall number of 90 W lesions and the impact on troponin levels, as the posterior wall is relatively thinner compared to the anterior wall. Anyway, the increased resistive heating phase correlated with vHPSD catheters allowed a reduced CRP increase after the procedure than in STD RF catheters, also in the presence of higher numerical values of hsTnI release. The significant difference in the decrease in serum potassium observed after catheter ablation with STD RF to vHPSD catheters could be attributed to the higher volume of 0.9% saline (NaCl) irrigation used with standard catheters. The larger fluid infusion with standard catheters can dilute serum potassium levels, creating a potential trend to hypokalemia. This finding suggests that vHPSD catheters may help mitigate procedure‐related potassium disturbances, reducing the risk of arrhythmias and improving procedural safety.

## 5 LIMITATIONS

Some limitations must be addressed to our research. First, our study is a single‐center study, so we cannot exclude the possibility of referral bias. Additionally, we evaluated the impact of vHPSD catheter use exclusively in a WACA approach, limiting the generalizability of our findings to more complex lesion sets. Long‐term biomarker evaluations were not performed, restricting insights into the sustained effects of the procedure. While the study focused on differences in heart failure biomarkers, post‐procedural clinical assessments were not conducted to detect the presence of subtle heart failure.

## 6 CONCLUSION

The use of a vHPSD catheter is associated with reduced fluid irrigation and a correspondingly smaller increase in BNP, a biomarker indicative of fluid overload and heart failure, regardless of the pre‐procedure BNP level. This effect could be particularly significant for patients with heart failure, as it may help minimize procedure‐related fluid overload.

## FUNDING INFORMATION

This research did not receive any specific grant from funding agencies in the public, commercial, or not‐for‐profit sectors.

## CONFLICT OF INTEREST STATEMENT

Authors declare no conflict of interests for this article.

## ETHICS STATEMENT

The study complies with the Declaration of Helsinki, and all participants provided written informed consent.

## PATIENT CONSENT STATEMENT

All participants provided written informed consent.

## PERMISSION TO REPRODUCE MATERIAL FROM OTHER SOURCES

All the material in the paper is original; there is no need for permission to reproduce material.

## Data Availability

The data that support the findings of this study are available from the corresponding author [AB] upon reasonable request.
